# HBV infection potentiates resistance to S-phase arrest-inducing chemotherapeutics by inhibiting CHK2 pathway in diffuse large B-cell lymphoma

**DOI:** 10.1038/s41419-017-0097-1

**Published:** 2018-01-19

**Authors:** Xinying Zhao, Xudong Guo, Libo Xing, Wenqin Yue, Haisen Yin, Miaoxia He, Jianmin Wang, Jianmin Yang, Jie Chen

**Affiliations:** 1 Department of Hematology, Changhai Hospital, Second Military Medical University, Shanghai, China; 2 Clinical and Translational Research Center of Shanghai First Maternity and Infant Health Hospital, Shanghai Key Laboratory of Signaling and Disease Research, Collaborative Innovation Center for Brain Science, School of Life Science and Technology, Tongji University, Shanghai, China; 3 Institute of Regenerative Medicine, East Hospital, Tongji University School of Medicine, Shanghai, China; 4 Department of Hematology, Jingzhou Central Hospital, Jingzhou Clinical Medical College, Yangtze University, Jingzhou, China; 5 Department of Pathology, Changhai Hospital, Second Military Medical University, Shanghai, China

## Abstract

A considerable number of diffuse large B-cell lymphoma (DLBCL) patients are infected with hepatitis B virus (HBV), which is correlated with their poor outcomes. However, the role of HBV infection in DLBCL treatment failure remains poorly understood. Here, our data demonstrated that HBV infection was closely associated with poorer clinical prognosis independent of its hepatic dysfunction in germinal center B-cell type (GCB type) DLBCL patients. Interestingly, we found that DLBCL cells expressing hepatitis B virus X protein (HBX) did not exhibit enhanced cell growth but did show reduced sensitivity to methotrexate (MTX) and cytarabine (Ara-C), which induced S-phase arrest. Mechanism studies showed that HBX specifically inhibited the phosphorylation of checkpoint kinase 2 (CHK2, a key DNA damage response protein). CHK2 depletion similarly conferred resistance to the S-phase arrest-inducing chemotherapeutics, consistent with HBX overexpression in DLBCL cells. Moreover, overexpression of wild-type CHK2 rather than its unphosphorylated mutant (T68A) significantly restored the reduced chemosensitivity in HBX-expressing cells, suggesting that HBV infection conferred resistance to chemotherapeutics that induced S-phase arrest by specifically inhibiting the activation of CHK2 response signaling in DLBCL.

## Introduction

Diffuse large B-cell lymphoma (DLBCL), which accounts for 30–40% of non-Hodgkin lymphoma (NHL), is an aggressive disease featuring heterogeneous genetic, phenotypic, and clinical characteristics^1^. The combination of rituximab and CHOP (cyclophosphamide, doxorubicin, vincristine, and prednisone) has dramatically improved the outcome in DLBCL patients, with the 3-year event-free survival ranging from 65 to 80%^2^. However, approximately 30–40% of DLBCL patients will develop relapsed or refractory, which remains the major cause of mortality^3^. Thus, it is imperative to investigate the mechanisms of relapse and refractory to develop better chemotherapy options.

Virus infection is considered as a critical inducer of various diseases, especially cancer. Epstein–Barr virus has been proven to associate with the occurrence and progression of nasopharyngeal carcinoma and NHL^4^. Hepatitis C virus and hepatitis B virus (HBV) are also able to promote NHL^5^. HBV has infected 350 million people worldwide and is responsible for 340,000 cases of liver cancer and 500,000 to 1.2 million liver-related deaths annually. In addition, epidemiologic investigations in HBV endemic areas, including China, South Korea, and Japan, show that HBV infection is more common in NHL patients compared with the general population (19.94% vs. 7.18%)^6–8^. Importantly, hepatitis B surface antigen (HBsAg)-positive DLBCL patients display poorer chemotherapy responses and shorter progression-free survival (PFS) and overall survival (OS) compared with HBsAg-negative patients as an independent prognostic factor in DLBCL^6,9^. These reports mainly focus on investigating the clinical correlation between HBV infection and poor prognosis of DLBCL, but the exact mechanisms remain unexplored. Hepatitis B virus X protein (HBX), has been reported to induce hepatocarcinogenesis by interacting with various signal transduction pathways, such as the SIRT^10^, Wnt/β-catenin^11^, signal transducer and activator of transcription^12^, and nuclear factor-κB pathway^13^ to induce hepatocarcinogenesis. The lymphotropic characteristic of HBV renders the HBX detectable in lymphoma tissue^14–17^. However, the role of HBX in causing progression and poor outcome in DLBCL patients has not been extensively studied.

Chemoresistance is the major cause of treatment failure, which results in relapsed and refractory DLBCL. The activation of the DNA damage response (DDR) allows cells self-repair to resist external damage via activating the downstream cyclins and apoptotic proteins, which results in chemoresistance^18^. DDR suppression is believed to sensitize tumor cells to chemotherapeutic treatments by causing cell death or senescence in the absence of checkpoints and efficient DNA repair^19–23^. On the other hand, DDR inhibition attenuates the cell cycle arrest that occurs during self-repair, which can reduce sensitivity to chemotherapeutic^24,25^. The role of DDR signals in the chemoresistance of DLBCL patients with HBV infection is still unclear.

After retrospectively analyzing 428 DLBCL patients, we found that HBV infection was closely associated with reduced responses to chemotherapy and poor OS and PFS of DLBCL patients, especially in germinal center B-cell type (GCB type) independent of its liver damage. Our results also showed that HBX conferred resistance to chemotherapeutics that induced S-phase arrest by specifically blocking the activation of checkpoint kinase 2 (CHK2) signaling *in vitro* and *in vivo*, which explained the poor outcome caused by HBV infection in DLBCL patients.

## Results

### HBV infection is correlated with poor prognosis of GCB-type DLBCL patients independent of its liver-damaging effects

We examined 428 DLBCL patients from January 2004 to July 2014 in Changhai Hospital (Shanghai, China) for HBsAg, and 93 cases were HBsAg positive. The HBV infection rate was much higher in DLBCL patients (21.7%) than that in the general population (7.18%)^6^. The clinical and pathological characteristics of DLBCL patients with or without HBV infection were shown in Table 1. The proportion of male patients with HBV infection (69.9%) was significantly higher than that without HBV infection (56.1%) (*P* = 0.017, Table 1). HBV-positive patients tended to be younger than HBV-negative patients (median age, 47.0 vs. 55.0, *P* = 0.032). There were no differences in cell of origin, Ki67 expression, lactate dehydrogenase (LDH), and proportions of rituximab included chemotherapy regimens between two groups, but HBV infection was closely associated with more advanced stages (*P* = 0.014, Table 1) and increased frequency of treatment failure (*P* = 0.028, Table 1), indirectly suggesting that HBV infection contributed to poor outcome.

**Table 1:** Characteristics of HBsAg-positive and HBsAg-negative subjects in Changhai Hospital (*n*=428)

Characteristics, *n* (%)	HBV (-), *n* = 335 (78.3%)	HBV (+), *n *= 93 (21.7%)	*P-*value
Gender			0.017
Male	188 (56.1%)	65 (69.9%)	
Female	147 (43.9%)	28 (30.1%)	
Median age	55.0	47.0	0.032
Stage			0.014
I+II	130 (38.8%)	23 (24.7%)	
III+IV	205 (61.2%)	70 (75.3%)	
Cell of origin			0.598
Non-GCB	190 (56.7%)	57 (61.3%)	
GCB	97 (29.0%)	25 (26.9%)	
Not available	48 (14.3%)	11 (11.8%)	
Liver function			0.002
Normal	320 (95.5%)	79 (84.9%)	
Abnormal	15 (4.5%)	14 (15.1%)	
LDH			0.082
Normal	231 (69.0%)	55 (59.1%)	
Elevated	104 (31.0%)	38 (40.9%)	
Median Ki67 expression	71.85%	70.00%	0.833
Chemotherapy outcome			0.028
CR+PR	246 (73.4%)	57 (61.3%)	
Relapse+NR	89 (26.6%)	36 (38.7%)	
Rituximab included			0.069
No	118 (35.2%)	43 (46.2%)	
Yes	217 (64.8%)	50 (53.8%)	

**Fig. 1:**
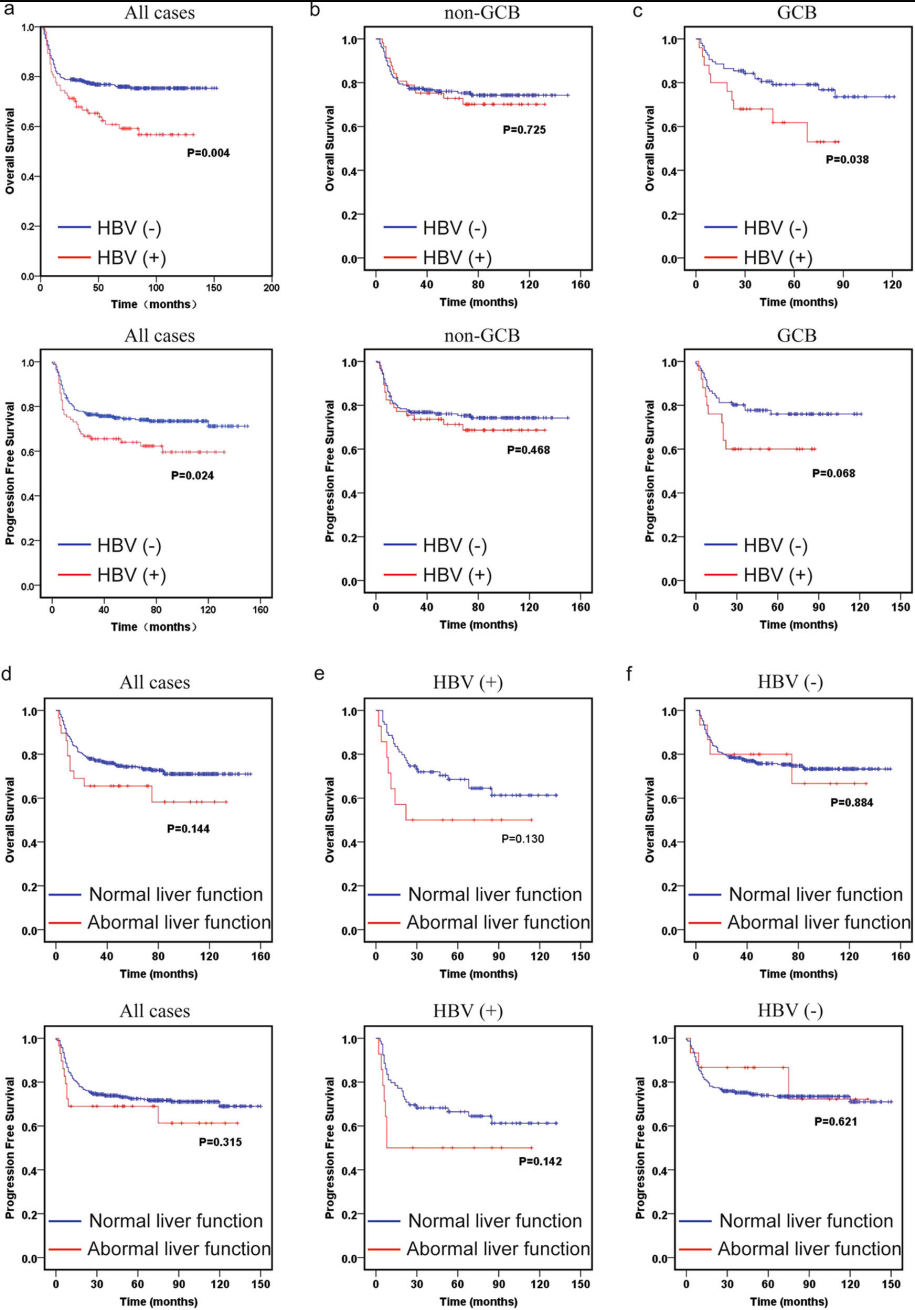
HBV infection associated with poor prognosis independent of its liver-damaging effects in GCB-type DLBCL **a-c** Kaplan-Meier analysis and log-rank test were performed to compare the OS and PFS of DLBCL patients with or without HBV infection in total cohort **a**, non-GCB type **b**, and GCB type **c**. **d-f** Kaplan–Meier analysis and log-rank test were performed to compare the OS and PFS of DLBCL patients of normal and abnormal liver function in total cohort **d**, HBV-positive **e**, and HBV-negative **f**, DLBCL patients

Kaplan–Meier analysis of the HBV-positive and HBV-negative cohorts revealed that HBV infection had a profound negative impact on the prognosis of DLBCL patients. Patients with HBV infection had significantly shorter OS and PFS than HBV-negative patients (Fig. 1a). After stratifying all the patients into GCB and non-germinal center B-cell (non-GCB) types, we found that DLBCL patients with HBV infection had significantly shorter OS in GCB type, but not non-GCB type, suggesting HBV infection mainly influenced the poor prognosis of GCB-type DLBCL patients (Figs. 1b, c). Increased hepatitis indicators, including alanine aminotransferase, aspartate aminotransferase, gamma-glutamyl transpeptidase, albumin, total bilirubin, and direct bilirubin, were more frequently observed in whole course of HBsAg-positive subjects (Supplementary Fig. 1a), in spite that they received anti-viral treatment before chemotherapy until 6 months after chemotherapy. We surprisingly found that abnormal liver function showed no significant impact on OS and PFS of the whole group, the HBV-positive cohort, or the HBV-negative cohort (Figs. 1d–f). In accordance with previous studies^14–17^, the HBX protein could be detected in lymphoid tissues of HBV-infected patients (Supplementary Fig. 1b). Our results thus indicated that HBV infection might reduce the OS and PFS of DLBCL patients by directly affecting the biological properties of DLBCL cells independent of its liver-damaging effects.

### HBX attenuates the proliferation inhibition of chemotherapeutics that induce S-phase arrest

**Fig. 2:**
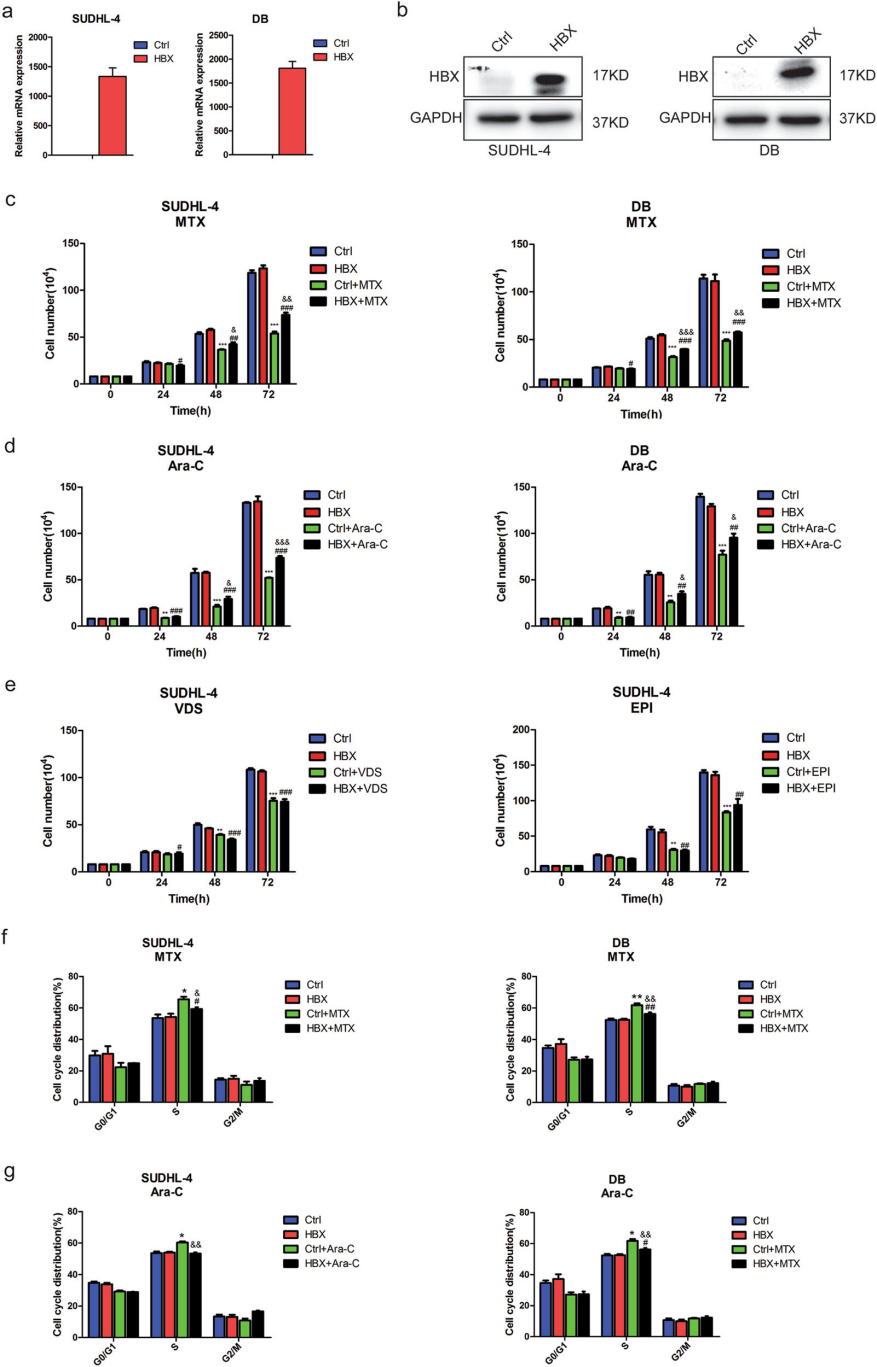
HBX attenuates the proliferation inhibition of chemotherapeutics that induce S-phase arrest **a,** **b** Ectopic overexpression of HBX in SUDHL-4 and DB cells was determined by qPCR **a**, and western blotting **b**. **c**, **d** Cell-counting assay for control or HBX-expressing SUDHL-4 and DB cells treated with MTX (4 ng/ml) or Ara-C (20 ng/ml); the cell numbers were measured at 24, 48, and 72 h. **e** Cell-counting assay for control or HBX-expressing SUDHL cells treated with VDS (0.6 ng/ml) or EPI (100 ng/ml); the cell numbers were measured at 24, 48, and 72 h. **f**, **g** Quantification of the cell cycle distributions in control and HBX-expressing SUDHL-4 and DB cells treated with MTX **f**, or Ara-C **g**, for 48 h. These results are shown as the mean ± SEM from triplicate experiments. “*” Represents Ctrl + agents versus Ctrl: *P < 0.05, **P < 0.01, ***P < 0.001. “#” Represents HBX + agents versus HBX: ^#^*P* < 0.05, ^##^*P* < 0.01, ^###^*P* < 0.001. “&” Represents HBX + agents versus Ctrl + agents: ^&^*P* < 0.05, ^&&^*P* < 0.01, ^&&&^*P* < 0.001

According to our clinical data analysis, the DLBCL patients with GCB-type HBV infection exerted shorter OS than that without HBV infection (Fig. 1c). We then focus on investigating the exact effects of HBX on the biological properties of GCB-type DLBCL cells. To analyze the effect of HBX on DLBCL cell growth, HBX was ectopically overexpressed in GCB-type DLBCL cell lines (SUDHL-4 and DB), which was verified by quantitative polymerase chain reaction (qPCR) and western blotting (Figs. 2a, b). However, HBX-expressing DLBCL cells did not exhibit enhanced proliferation or differences in cell cycle distribution compared with control cells (Supplementary Figs. 2a and b). There was also no significant difference in the expression of cell cycle-related proteins upon exogenous expression of HBX compared with control cells (Supplementary Figs. 2c and d). Neither control cells nor HBX-expressing cells exhibited obvious apoptosis, as evaluated by flow cytometry (Supplementary Fig. 2e). These data suggested that HBX had no significant effect on the proliferation, cell cycle distribution, or apoptosis of DLBCL cells.

We further sought to investigate the effect of HBX on the response to chemotherapeutics. We tested the growth of control and HBX-expressing cells treated with the chemotherapeutic agents methotrexate (MTX), cytarabine (Ara-C), vindesine (VDS), and epirubicin (EPI) at a concentration of the IC50 value for 24, 48, and 72 h. Compared with control cells, SUDHL-4 and DB cells overexpressing HBX showed enhanced resistance to MTX and Ara-C after treatment for 48 and 72 h (Figs. 2c, d), whereas there was no significant difference in proliferation inhibition upon treatment with VDS and EPI (Fig. 2e). Furthermore, the cell cycle distributions analysis of control and HBX-expressing cells treated with or without MTX or Ara-C, which were characterized by inducing S-phase arrest, showed that HBX overexpression significantly attenuated the S-phase arrest in both SUDHL-4 and DB cells upon treatment with MTX and Ara-C (Figs. 2f, g). These findings indicated that HBX prevented the cytotoxicity and impaired the S-phase arrest of chemotherapeutics, which might explain why HBV infection cause poor outcome in DLBCL patients.

### HBX specifically inhibits CHK2 phosphorylation

**Fig. 3:**
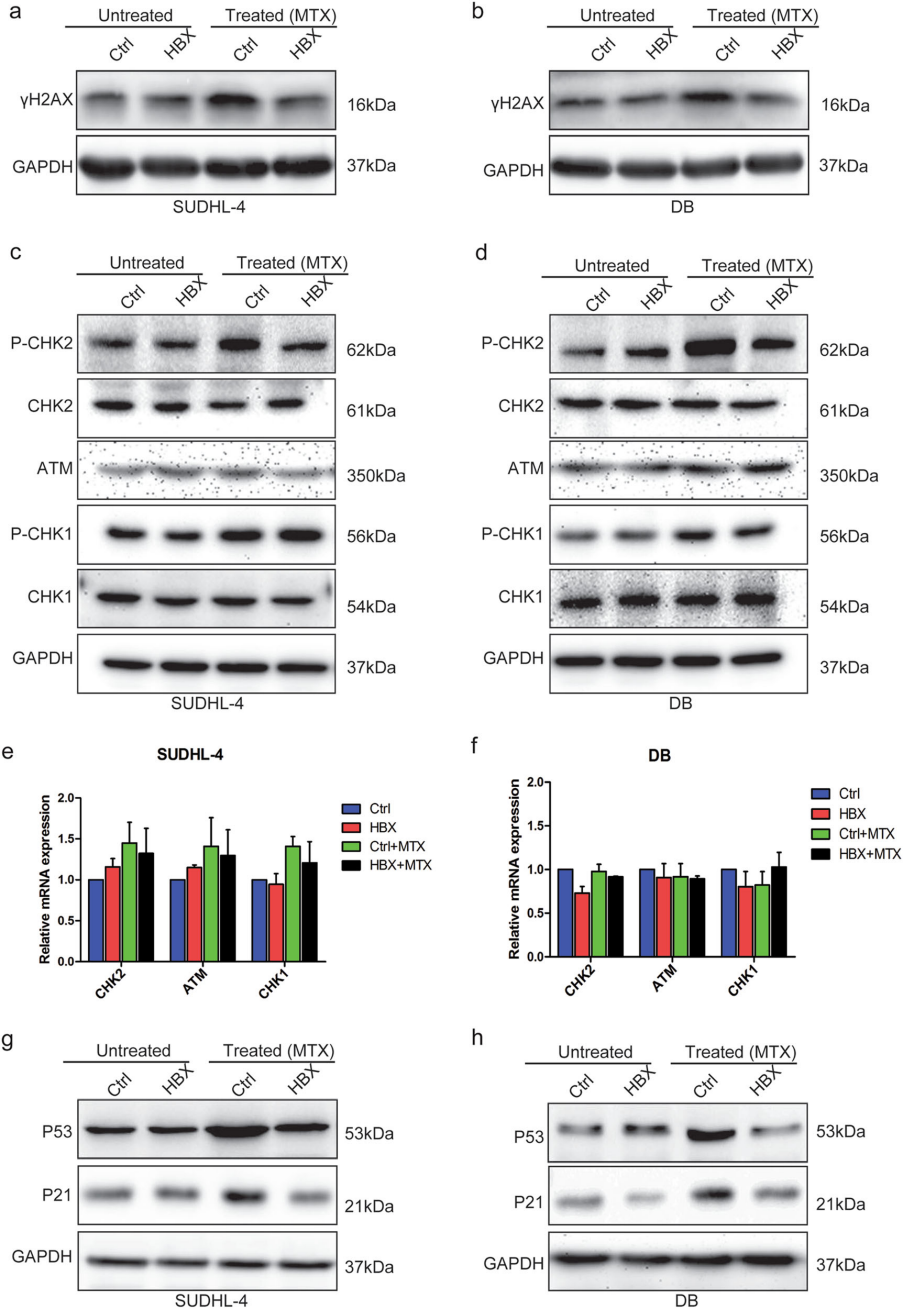
HBX specifically inhibits CHK2 phosphorylation **a**, **b** Western blotting for γH2AX in control and HBX-expressing SUDHL-4 **a**, and DB **b**, cells with or without MTX treatment (4 ng/ml) for 48 h. **c**, **d** Western blotting for DDR proteins (P-CHK2/CHK2/ATM and P-CHK1/CHK1) in control and HBX-expressing SUDHL-4 **c**, and DB **d**, cells with or without MTX treatment (4 ng/ml) for 48 h. **e**, **f** The mRNA levels of DDR proteins (ATM, CHK2, and CHK1) were largely unchanged in control and HBX-expressing SUDHL-4 **e**, and DB **f**, cells with or without MTX treatment. **g,** **h** The expression of the P-CHK2 downstream genes P53 and P21 in control and HBX-expressing SUDHL-4 **g**, and DB **h**, cells with or without MTX treatment. The results are shown as the mean ± SEM from triplicate experiments. GAPDH was used as a loading control

DDR has been reported to be intimately correlated with chemoresistance^26^. Phosphorylation of H2AX at Ser139 (γH2AX) is one of the early response to DNA damage, which is considered as a sensitive biomarker for DDR^27,28^. We found that HBX-expressing cells presented lower expression of γH2AX than control cells after treated with MTX, indicating that HBX expression attenuated the chemotherapeutic-induced DNA damage in DLBCL cells (Figs. 3a, b). To gain mechanistic insight into the molecules potentially involved in the decreased chemosensitivity resulting from HBV infection, alterations in two major DDR pathways ataxia-telangiectasia mutated (ATM)/CHK2, and ATM and Rad-3 related (ATR)/checkpoint kinase 1 (CHK1) were assessed. The expression level of P-CHK1 was increased after treatment with MTX for 48 h in both control and HBX-expressing cells, but neither the baseline nor the post-treatment level of P-CHK1 was affected by overexpressing HBX. Unexpectedly, the expression level of P-CHK2 in control cells was markedly increased to a greater extent than that in HBX-expressing cells after treatment with MTX for 48 h, whereas no significant difference was observed in the baseline P-CHK2 level between control and HBX-expressing cells (Figs. 3c, d). Both the mRNA and protein levels of CHK1, CHK2, and ATM (an upstream kinase of CHK2) were largely unchanged, indicating that HBX might specifically inhibit the phosphorylation of CHK2 (Figs. 3e, f). Activated CHK2 signaling induced cell cycle arrest through activating P53 followed by P21^29^. Our results showed that the expression levels of P53 and P21, consistent with the expression level of P-CHK2, were less increased in HBX-overexpressing cells after treatment with MTX than that in control cells (Figs. 3g, h). Together, these results indicated that HBX overexpression appeared to specifically inhibit CHK2 phosphorylation caused by MTX treatment in DLBCL cells.

### CHK2 phosphorylation rescues chemosensitivity in HBX-expressing cells

**Fig. 4:**
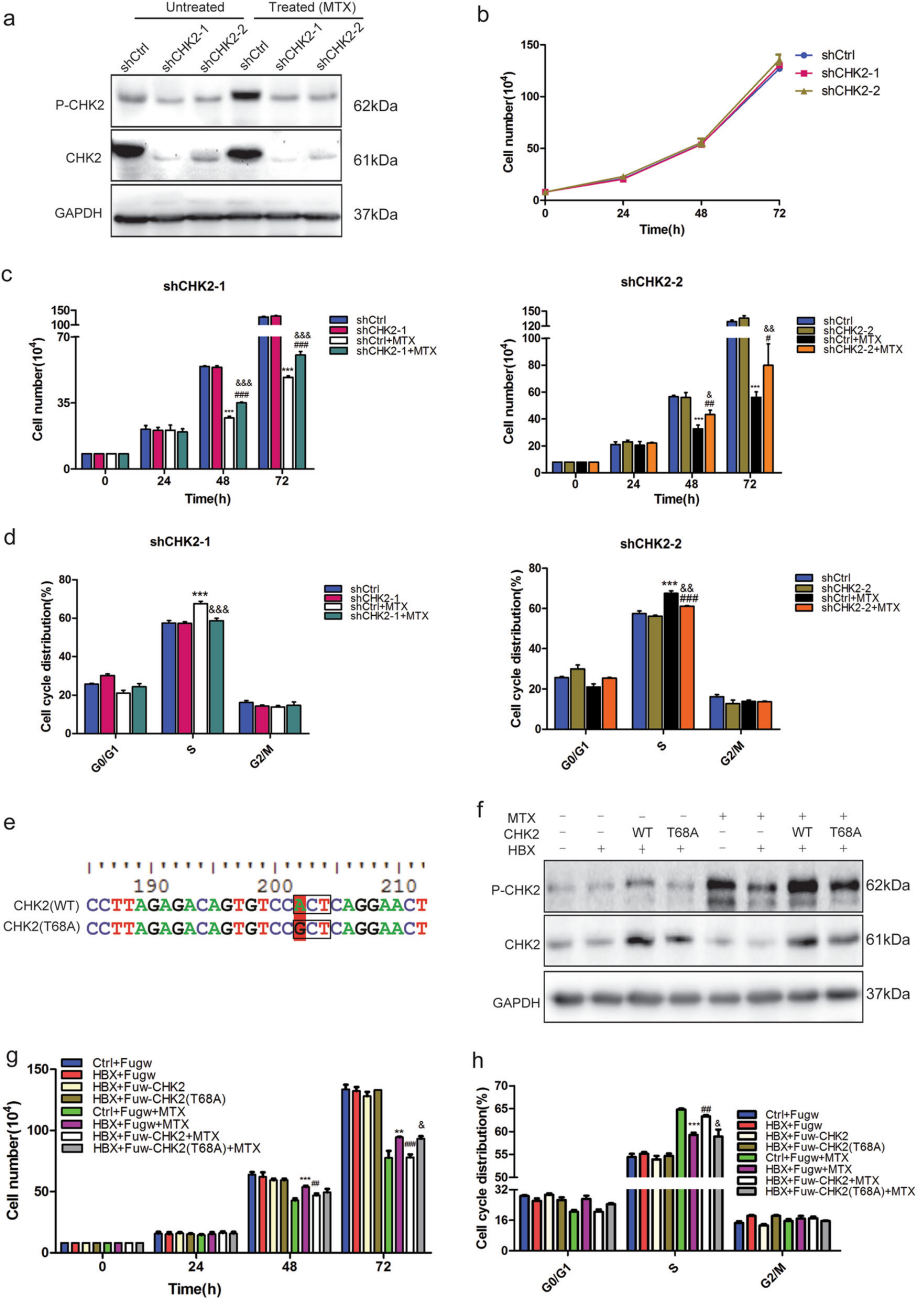
CHK2 phosphorylation rescues chemosensitivity in HBX-expressing cells **a** The expression of CHK2 and P-CHK2 was measured by western blotting upon CHK2 depletion by two independent shRNAs with or without MTX treatment. **b** Depletion of CHK2 showed no significant effect on the proliferation of DLBCL cells. **c** Cell-counting assays for control, shCHK2-1 and shCHK2-2 SUDHL-4 cells with or without MTX (4 ng/ml) treatment at 24, 48, and 72 h. **d** Quantification of the cell cycle distributions of control and HBX-expressing cells with or without MTX treatment for 48 h. The results are shown as the mean ± SEM from triplicate experiments. “*” Represents shCtrl + MTX versus shCtrl: **P < 0.01, ***P < 0.001. “#” Represents shCHK2 + MTX versus shCHK2: ^##^*P* < 0.01, ^###^*P* < 0.001. “&” Represents shCHK2 + MTX versus shCtrl + MTX: ^&^*P* < 0.05, ^&&^*P* < 0.01, ^&&&^*P* < 0.001. **e** The schematic diagram of wild-type and mutant (T68A) CHK2. **f** Western blotting analysis for CHK2 and P-CHK2 in control and HBX-expressing cells transfected with Fuw (empty vector), Fuw-CHK2 (WT), or Fuw-CHK2 (T68A) with or without MTX treatment. GAPDH was used as a loading control. **g,** **h** Cell counting **g**, and cell cycle distribution analysis **h**, of the indicated groups with or without MTX treatment. The results are shown as the mean ± SEM from triplicate experiments. “*” Represents HBX + MTX versus Ctrl + MTX: **P < 0.01, ***P < 0.001. “#” Represents HBX + CHK2(WT) + MTX versus HBX + MTX: ^##^*P* < 0.01, ^###^*P* < 0.001. “&” Represents HBX + CHK2(T68A) + MTX versus HBX + CHK2(WT) + MTX: ^&^*P* < 0.05

To investigate the functional role of P-CHK2 in chemoresistance, we attempted to downregulate CHK2 in SUDHL-4 cells using short hairpin RNA (shRNA). CHK2 depletion led to reduced expression of both P-CHK2 and CHK2 (Fig. 4a). The increase in P-CHK2 was less pronounced in shCHK2-1 and shCHK2-2 cells with MTX treatment compared with control cells (Fig. 4a). We further found that CHK2-depleted cells exhibited normal cell growth compared to control cells, consistent with previous reports (Fig. 4b)^30^. CHK2-depleted cells displayed a modest proliferation decrease and S-phase increase after MTX treatment for 48 h, which simulated the effects of HBX overexpression on proliferation and the proportion of cells in S-phase (Figs. 4c, d). As downregulation of CHK2 could result in enhanced chemoresistance after MTX treatment, we tried to reverse the reduced chemosensitivity of HBX-expressing cells by overexpressing wild-type CHK2 (WT) or an unphosphorylated CHK2 mutant (T68A) (Fig. 4e). An increase in the CHK2 level was detected after overexpressing both CHK2 (WT) and CHK2 (T68A), whereas increased expression of P-CHK2 was only observed after CHK2 (WT) overexpression (Fig. 4f). As expected, overexpressing CHK2 (WT) rather than mutant CHK2 (T68A) reversed the reduced proliferation inhibition and decreased S-phase arrest caused by HBX overexpression after MTX treatment (Figs. 4g, h). These results indicated that CHK2 could overcome HBX-induced chemoresistance and that this effect was dependent on its phosphorylation.

### HBX confers resistance to chemotherapy *in vivo*

**Fig. 5:**
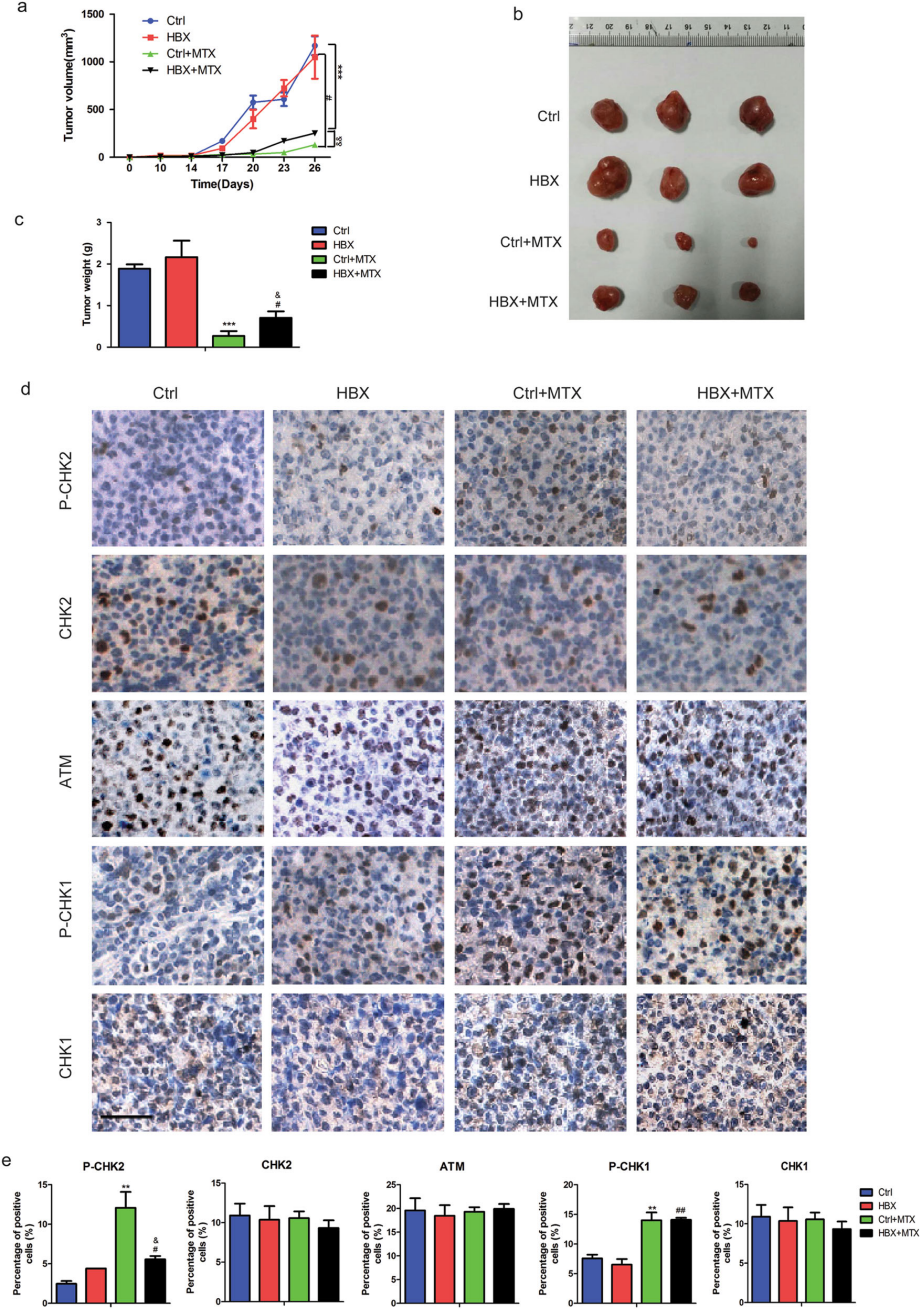
HBX confers resistance to chemotherapy *in vivo* **a** Tumor volumes were measured every 3 days after day 14 post-injection in the indicated groups (*n* = 3 per group). **b**, **c** Representative images **b**, and weight statistics **c**, of tumors isolated from the mice for one of the batches at day 26 post-injection. **d** Immunohistochemical analysis of tumor sections from four groups using the indicated antibodies (P-CHK2, CHK2, ATM, P-CHK1, and CHK1). Scale bar, 25 μm. **e** Histogram statistics for the cells positive for the indicated proteins are shown. “*” Represents Ctrl + MTX versus Ctrl: **P < 0.01, ***P < 0.001. “#” Represents HBX + MTX versus HBX: ^#^*P* < 0.05,^##^ *P* < 0.01. “&” Represents HBX + MTX versus Ctrl + MTX: ^&^*P* < 0.05,^&&^*P* < 0.01

To further assess HBV infection as a mediator of chemoresistance, we examined the inhibition effect of MTX on control and HBX-expressing cells *in vivo*. Control and HBX-overexpressing DB cells were grafted into the nonobese diabetic-severe combined immunodeficiency disease (NOD-SCID) mice. Two weeks after injection, the mice were treated with MTX (10 mg/kg by intraperitoneal injection every 3 days), and tumor sizes were measured (Fig. 5a). MTX treatment significantly reduced the tumor burden of mice injected with control cells compared with untreated mice (Figs. 5b, c). However, the tumor burden in mice injected with DB cells expressing HBX was not reduced as effectively as that in mice injected with control cells (Figs. 5b, c). The tumor specimens were then subjected to immunohistochemical staining to analyze the expression of DDR proteins, and our data showed that the expression level of P-CHK2 was mildly increased in tumors derived from HBX-expressing cells compared with those derived from control cells, whereas the increase extent of P-CHK1 was not affected by HBX overexpression (Figs. 5d, e). Consistent with the results *in vitro*, the expression levels of CHK2, ATM, and CHK1 were largely unchanged in both control and HBX-expressing cells with or without MTX treatment (Figs. 5d, e). These *in vivo* findings showed that HBV infection was a potent mediator of resistance to chemotherapeutics that induce S-phase arrest.

## Discussion

Large-scale epidemiological investigations have suggested that HBV infection is positively correlated with the onset and progression of NHL. A large cohort study enrolling 603,585 individuals (53,045 HBV positive) in Korea indicated that the HBV-positive population had an increased risk of developing DLBCL throughout 14 years of follow-up^31^. It has also been reported that HBsAg-positive DLBCL patients exhibit more advanced disease stages, lower response to chemotherapy, and worse outcomes compared with HBsAg-negative DLBCL patients^5,9^. HBV-infected DLBCL patients are more likely to experience liver damage, while it is still conflicted that whether hepatic dysfunction serves as a prognosis factor for DLBCL during chemotherapy^6,9^. Our data demonstrated that HBsAg-positive DLBCL patients especially GCB type had lower chemotherapy efficacy and poorer outcomes than HBsAg-negative patients and that HBV-induced chemoresistance, but not hepatic dysfunction, contributed to the poor outcome.

Chemoresistance is widely known as a major cause of treatment failure^18,32–34^. HBV-infected DLBCL patients are more likely to exhibit advanced disease stages, which often preclude the first-line chemotherapies, including CHOP and R-CHOP. Our results showed that overexpression of the HBX protein resulted in resistance to the S-phase arrest-inducing chemotherapeutics (MTX and Ara-C) rather than two other drugs (VDS and EPI) in DLBCL cells, which was further confirmed by the impaired S-phase arrest in HBX-overexpressing DLBCLs treated with MTX or Ara-C. Thus, our findings indicated that HBV infection might result in lower sensitivity to MTX- or Ara-C-based second-line chemotherapy regimens including ESHARP (etoposide, Ara-C and prednisone), MA (MTX and Ara-C), DHAP (cisplatin, Ara-C and prednisone,and CODOX-MTX (cyclophosphamide, VDS, doxorubicin, and HD-MTX). Further investigations of autologous hematopoietic hepatocyte transplantation^35^ and other intensive chemotherapies, including R-EPOCH (rituximab, etoposide, cyclophosphamide, doxorubicin, vincristine, and prednisone)^36,37^, which have shown favorable effects in the treatment of aggressive DLBCL, might be of great benefit to DLBCL patients infected with HBV. Several studies have indicated that HBV infection results in the onset and progression of hepatocellular carcinoma because of its hepatotropic peculiarity^38,39^. HBV-DNA is consisted of four segments (S, P, C, and X). HBX, coded by HBV-X segment, is considered as a small “transcriptional activator” that stimulates transcription by activating Ras–Raf–mitogen-activated protein kinase (MAPK) signaling cascade and promotes cell proliferation by deregulating cell cycle checkpoint controls, then contributes to viral carcinogenesis^40,41^. HBX was also reported to promote hepatocarcinogenesis by regulating various signals^10–12.^ However, our present data found that HBX had no effects on the cell growth, cell cycle distribution, or apoptosis of DLBCL cells, but reduced the chemosensitivity to drugs that induce S-phase arrest. The roles of other HBV segments in the DLBCL cell property needed to be further explored. Collectively, our study indicated a new function of HBX in regulating DLBCL cell chemoresistance and suggested that HBsAg-positive DLBCL patients would benefit less from MTX- or Ara-C-based chemotherapy than HBsAg-negative ones.

Much work has demonstrated that DDR is closely related to chemoresistance^18,42,43^. ATM/CHK2 and ATR/CHK1, two key pathways in DDR signaling, are activated upon DNA damage, leading to cell cycle arrest^19–23^. Our results showed that both MTX- and Ara-C-induced DNA damage dramatically activated CHK2 signaling and caused S-phase arrest to inhibit DLBCL cell growth. HBX overexpression significantly attenuated the activation of CHK2 signaling induced by S-phase arrest drugs, together with its downstream proteins P53 and P21, but did not affect the CHK1 pathway. CHK2 knockdown mimicked HBX-induced resistance to the S-phase arrest-inducing agents, and CHK2 overexpression overcame the resistance caused by HBX overexpression, indicating that CHK2 might serve as a potential mediator of HBX-induced chemoresistance and its de-phosphorylation caused by HBX impaired the S-phase arrest via inhibiting the downstream P53-P21 pathway in DLBCL. Furthermore, our present data showed that the chemosensitivity of HBX-expressing cells could be rescued by overexpressing wild-type CHK2, but not the unphosphorylated CHK2 mutant (T68A). HBX has previously been reported to modulate the phosphorylation of phosphatidylinositol 3 kinase/AKT and MAPK/extracellular signal-regulated kinase signals in liver and pancreatic cancer^44,45^ and to activate CHK2 phosphorylation to delay the cell cycle in hepatocarcinogenesis^46^, suggesting that the direct regulation of protein phosphorylation is an important mechanism for HBX in carcinogenesis. Our findings first indicated that HBX specifically inhibited CHK2 phosphorylation, which conferred resistance to chemotherapeutics that induced S-phase arrest in DLBCL.

Evidence has shown that combining cytotoxic chemotherapeutics with pharmacological CHK2 inhibitors can prevent damaged cancer cells from arresting and improve therapeutic efficacy^25,47,48^. CHK2 inhibitors have already entered phase I & II clinical trials^48,49^. However, it has also been reported that CHK2 depletion reduces the chemosensitivity of various DNA-damaging agents including MTX in the treatment of Burkitt lymphoma^24^. High expression of CHK2 also results in a better response to platinum-based chemotherapy in ovarian cancer^25^. Our results showed that HBV infection caused resistance to S-phase arrest-inducing drugs in DLBCL by suppressing CHK2 phosphorylation, suggesting that HBV-infected DLBCL patients might not benefit from the use of CHK2 inhibitors.

In summary, our study uncovered the association between HBV infection and resistance to MTX- or Ara-C-based chemotherapy mediated by the inhibition of CHK2 response signaling, which provides new insight into the role of HBV in the chemoresistance of DLBCL patients. Therefore, HBV-infected DLBCL patients may benefit less from the use of S-phase arrest-inducing chemotherapeutics or CHK2 inhibitors. Further prospective investigations utilizing HBX-directed targeted therapy may help manage this challenge.

## Materials and methods

### Patients

We collected clinical data (name, gender, stages, international prognostic index (IPI) score, cell of origin, Ki67 expression, LDH, chemotherapy regiments, therapeutic evaluation, HBsAg status, liver indicator, OS, and PFS) of 428 cases DLBCL patients with or without HBV infection from January 2004 to July 2014 in Changhai Hospital, Shanghai, China. This study was approved by the institutional review board of the Changhai Hospital. The diagnosis of B-cell lymphoma used current World Health Organization (WHO) classification criteria. HBV infection status was determined by HBsAg. Stage criteria were based on the Ann Arbor staging criteria. The treatment efficacy was evaluated according to WHO curative standard.

### Cell culture and chemotherapeutics

Human 293T cells and DLBCL cell lines (SUDHL-4 and DB) were purchased from the American Type Culture Collection (ATCC) and cultured in Dulbecco’s modified Eagle’s medium containing 10% fetal bovine serum (Gibco) at 37 °C with 5% CO_2_ in a humidified incubator. The following chemotherapeutics were used: MTX (Pfizer, Shanghai, China), Ara-C (Pharmacia, Shanghai, China), EPI (Hisun, Hangzhou, China), and VDS (Minsheng, Hangzhou, China).

### Plasmid construction and stable transfection

The DNA fragments of HBX gene cloned from HepG2.2.15 cells were inserted into the pLVX-3× flag-IRES-ZsGreen1 vector, and the CHK2 DNA fragments cloned from SUDHL-4 cells were inserted into the Fuw vector. The CHK2 shRNAs were inserted into the pLKO.1 vector. Plasmid transfection was performed according to protocols supplied with the X-tremeGENE (Roche, Indianapolis, USA). Viral supernatant harvested 48 h after transfection was used to infect DLBCL cells in the presence of polybrene (8 μg/ml). Infected cells were selected with puromycin (Invitrogen, Carlsbad, CA, USA) at a final concentration of 2 μg/ml. The primers are shown in Supplementary Table S1.

### Cell growth assays and cell cycle analysis

Cells (8 × 10^4^) were seeded in a 12-well plate, and the indicated amounts of chemotherapeutics were added. Cell numbers were measured by Cell Counter Star after 24, 48, and 72 h. The cells were harvested at 48 h and fixed in 1 ml of pre-cooled 70% ethanol for at least 8 h. The cells were stained with propidium iodide/RNase (Beyotime, Shanghai, China) at 37 °C for 30 min in the dark. Cell cycle distributions were evaluated by flow cytometry using a FACS Calibur (Becton Dickinson Biosciences, Franklin Lakes, NJ, USA) and analyzed by FlowJo software.

### Western blotting and immunohistochemistry

Western blotting and immunohistochemistry were performed according to standard procedures^50^. The following antibodies were used: anti-HBX (ab39716, Abcam, Cambridge, MA, USA), anti-P-CHK2 (2197S, Cell Signaling Technology, Danvers, MA, USA), anti-CHK2 (ab47433, Abcam), anti-ATM (ab199726, Abcam), anti-P21 (ab7960, Abcam), anti-P53 (BS1913, Bioworld, Minnesota, USA), anti-γH2AX (ab26350, Abcam), anti-P-CHK1 (2348S, Cell Signaling Technology), anti-CHK1 (10362-1-AP, Proteintech), anti-CDK2 (SC-6248, Santa Cruz Biotechnology, CA, USA), anti-Cyclin D1 (SC-718, Santa Cruz Biotechnology), anti-GAPDH (SC-47724, Santa Cruz Biotechnology), HRP-Ms (#7074, Cell Signaling Technology), and HRP-Rb (#7076, Cell Signaling Technology).

### Quantitative RT-PCR (qPCR)

Total RNA was extracted from the cells using RNAiso (9109, Takara, Japan). First-strand complementary DNA synthesis was performed using the PrimeScript^TM^ RT reagent kit (RR037A, Takara). The qPCR analysis was performed using SYBR^®^ Premix Ex Taq TM (RR420A, Takara) with an Agilent Stratagene Mx3000P instrument. The GAPDH gene was employed as an internal control. The primer sequences used in this study are shown in Supplementary Table S2.

### Tumor xenografts

Five-week-old NOD-SCID mice were purchased from the National Resource Center for Rodent Laboratory Animals of China. Control and HBX-expressing DB cells (1 × 10^7^ each), suspended in 100 μl of one part Matrigel and two parts DMEM, were subcutaneously injected into the left and right thigh of mice, respectively. On day 14 after tumor cells injection, the mice were monitored to assess the tumor volume using the formula 1/2 (length × width^2^) and injected intraperitoneally with MTX (10 mg/kg) every 3 days until day 26. The mice were sacrificed on day 26 post-injection. All experiments were carried out as approved by the Institutional Animal Care and Use Committee of Tongji University.

### Statistical analysis

The Kaplan–Meier method and log-rank test were used to generate and compare the OS and PFS between different groups. We used the chi-square test and non-parametric test to compare categorical variables. The statistics were prepared using Excel and GraphPad Prism 5 software. The experiments were performed in triplicate and all data were presented as the mean ± standard error of mean (SEM). Normally distributed groups were compared by two-tailed Student’s *t-*test. *P* < 0.05 was considered statistically significant.

## Supplementary Information

Supplementary information
