# Supplementary material for: HBV infection potentiates resistance to S-phase arrest-inducing chemotherapeutics by inhibiting CHK2 pathway in diffuse large B-cell lymphoma
Source: Cell Death Dis. 2018 Jan 19;9(2):61. doi: 10.1038/s41419-017-0097-1 (PMC5833392; doi:10.1038/s41419-017-0097-1)
Supplement: Supplementary file 1 — Supplementary information [file 41419_2017_97_MOESM1_ESM.docx]

**
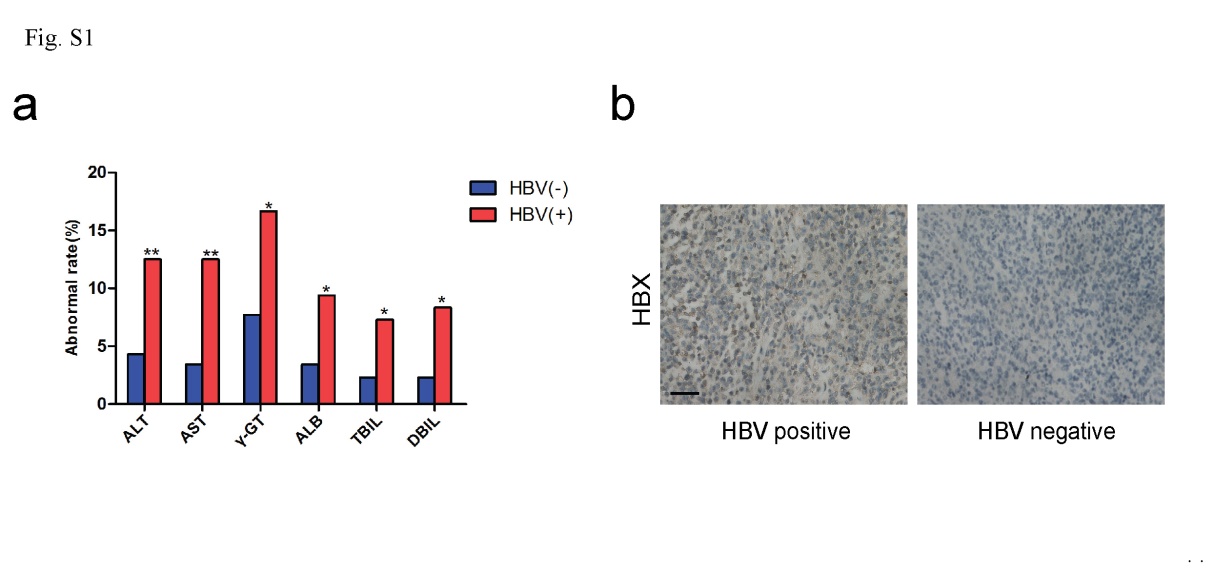
**

**Supplementary Figure 1** HBV infection had a negative impact on liver function. (**a)** Chi-square test was performed to compare the differences in liver function indicators (ALT, AST, γ-GT, ALB, TBIL and DBIL) between the HBV-positive and HBV-negative groups. (**b**) Immunohistochemical analysis of tumor sections from HBV-positive and HBV-negative patients using HBX antibody. Scale bar, 25 μm.


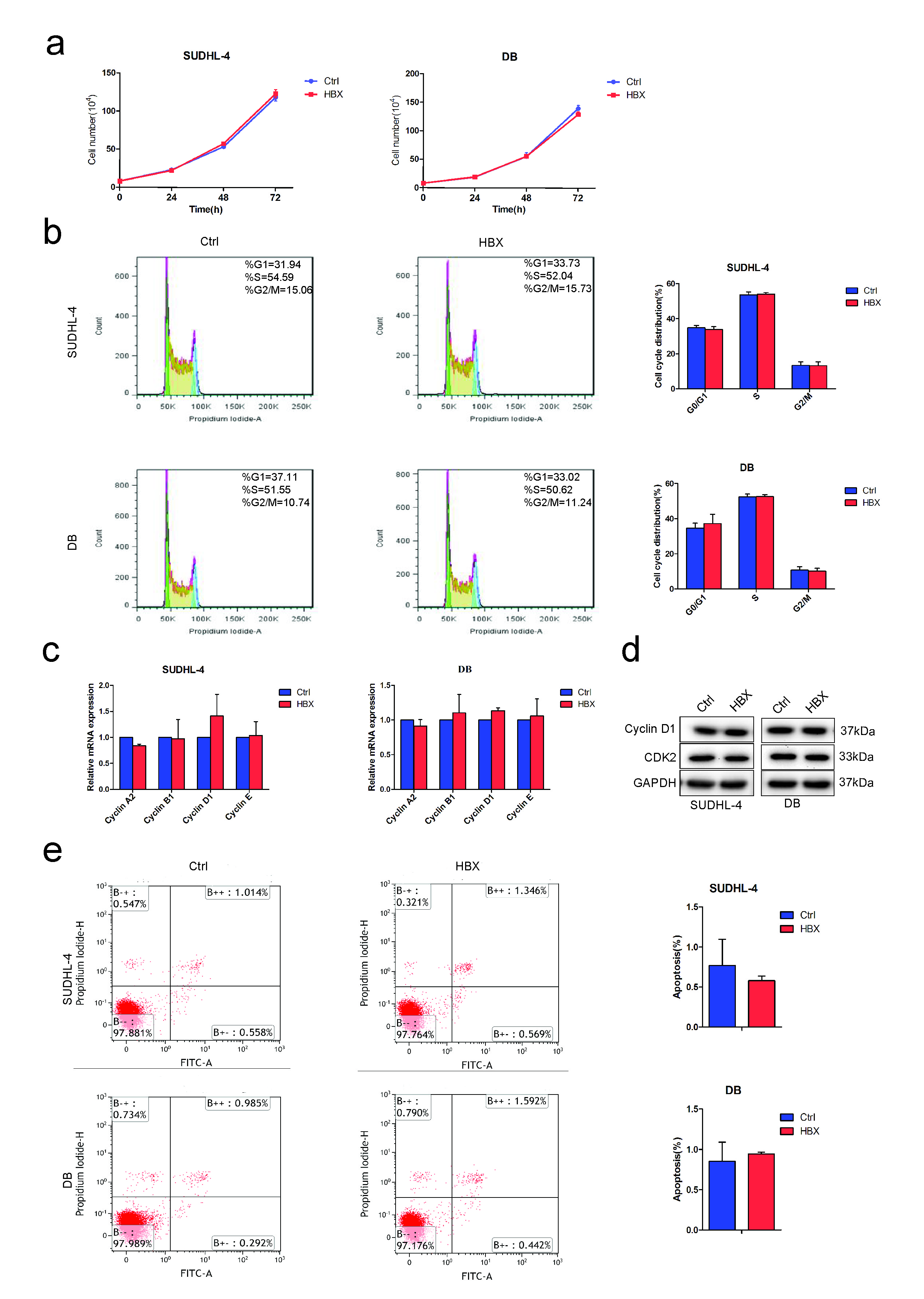


**Supplementary Figure 2** HBX shows no significant effects on the cell proliferation and apoptosis of DLBCL cells. (**a**) The effect of HBX on the proliferation of SUDHL-4 (left) and DB (right) cells. The numbers of control and HBX-expressing cells were counted after 24 h, 48 h, and 72 h. (**b**) Representative images and quantification of the cell cycle distributions in control and HBX-expressing SUDHL-4 (top) or DB (bottom) cells. (**c** and **d**) The mRNA (**c**) and protein (**d**) expression of cell cycle-related genes (cyclin A2, cyclin B1, cyclin D1, cyclin E and CDK2) in control and HBX-expressing cells. GAPDH was used as loading control. (**e**) Representative images and quantification of apoptosis in control and HBX-expressing cells. The results are shown as the mean ±SEM from triplicate experiments.

**Table S1** Primers used for vector construction.

| Plvx-HBX-F | CCGCTCGAGGCCACCATGGCTGCTAGGCTGTGCTGC |
| --- | --- |
| Plvx-HBX-R | TCCGAAGATCTGGCAGAGGTGAAAAAGTTGCATG |
| shCHK2-1-F | CCGGGGAGAGGTAAAGCTGGCTTTCCTCGAGGAAAGCCAGCTTTACCTCTCCTTTTTG |
| shCHK2-1-R | AATTCAAAAAGGAGAGGTAAAGCTGGCTTTCCTCGAGGAAAGCCAGCTTTACCTCTCC |
| shCHK2-2-F: | CCGGGGCTGTGCAGTACCTTCATGACTCGAGTCATGAAGGTACTGCACAGCCTTTTTG |
| shCHK2-2-R | AATTCAAAAAGGCTGTGCAGTACCTTCATGACTCGAGTCATGAAGGTACTGCACAGCC |
| Fuw-CHK2-F | CACAGACCGGTATGTCTCGGGAGTCGGAT |
| Fuw-CHK2-R | CACAGGAATTCTCACAACACAGCAGCACACACAGC |
| Fuw-CHK2(T68A)-F | CCTTAGAGACAGTGTCCGCTCAGGAACTCTATTCT |
| Fuw-CHK2(T68A)-R | AGAATAGAGTTCCTGAGCGGACACTGTCTCTAAGG |

**Table S2** Primers used in qPCR assays.

| ATM-qpcr-F | GGCTATTCAGTGTGCGAGACA |
| --- | --- |
| ATM-qpcr-R | TGGCTCCTTTCGGATGATGGA |
| CHK1-qpcr-F | CCAGTAAACAGTGCTTCTAG |
| CHK1-qpcr-R | TCTTCAGGAAGTGTCTCTTGC |
| CHK2-qpcr-F | GTCATCTCAAGAAGAGGACT |
| CHK2-qpcr-R | GAGCTGTGGATTCATTTTCC |
| HBX-qpcr-F | TTCTCCGCCTGTTCTACCGA |
| HBX-qpcr-R | TGAAAGTCCAAGAGTCCTCTTATGC |
